# A mitochondrial division inhibitor, Mdivi-1, inhibits mitochondrial fragmentation and attenuates kainic acid-induced hippocampal cell death

**DOI:** 10.1186/s12868-016-0270-y

**Published:** 2016-06-10

**Authors:** Hwajin Kim, Jong Youl Lee, Keon Jae Park, Won-Ho Kim, Gu Seob Roh

**Affiliations:** Department of Anatomy and Convergence Medical Science, Institute of Health Sciences, Gyeongsang National University School of Medicine, 15, 816 Beon-gil, Jinju-daero, Jinju, Gyeongnam 660-751 Republic of Korea; Division of Metabolic Diseases, Center for Biomedical Sciences, Korea National Institute of Health, Osong, Republic of Korea

**Keywords:** Drp1, Mitochondrial fission, Neuroinflammation, Neuronal cell death

## Abstract

**Background:**

Kainic acid (KA)-induced excitotoxicity promotes cytoplasmic calcium accumulation, oxidative stress, and apoptotic signaling, leading to hippocampal neuronal death. Mitochondria play a critical role in neuroinflammation and the oxidative stress response. Mitochondrial morphology is disrupted during KA-induced seizures; however, it is not clear whether mitochondrial fission or fusion factors are involved in KA-induced neuronal death.

**Results:**

We investigated the effect of Mdivi-1, a chemical inhibitor of the mitochondrial fission protein Drp1, on mitochondrial morphology and function in KA-injected mice. Mdivi-1 pretreatment significantly reduced seizure activity and increased survival rates of KA-treated mice. Mdivi-1 was protective against mitochondrial morphological disruption, and it reduced levels of phosphorylated Drp1 (Ser616) and Parkin recruitment to mitochondria. By contrast, levels of mitochondrial fusion factors did not change. Mdivi-1 also reduced KA-induced neuroinflammation and glial activation.

**Conclusions:**

We conclude that inhibition of mitochondrial fission attenuates Parkin-mediated mitochondrial degradation and protects from KA-induced hippocampal neuronal cell death.

**Electronic supplementary material:**

The online version of this article (doi:10.1186/s12868-016-0270-y) contains supplementary material, which is available to authorized users.

## Background

Kainic acid (KA) is a powerful neurotoxic analog of glutamate that induces excitotoxic neuronal death in the hippocampus [1, 2] through the accumulation of multiple cellular stress responses [3]. Mitochondria are cellular organelles that play a crucial role in ATP energy production, calcium homeostasis, and inflammation in the central nervous system. Mitochondrial dysfunction is found in many neurodegenerative diseases, and pathology is related to mitochondrial calcium overload, synaptic energy failure, inflammation, and oxidative stress [4, 5]. Mitochondrial dysfunction and structural damage have also been reported in epileptic seizures in humans and animal models [6–8].

Mitochondria are highly dynamic structures that consistently change in size and shape. Mitochondrial fission segregates dysfunctional mitochondria for degradation, and fusion results in the sharing of newly synthesized RNA and proteins with pre-existing mitochondria to maintain healthy mitochondrial pools [9]. In neurons, mitochondrial fission facilitates movement of mitochondria to distal axons to supply ATP energy for proper synaptic function and prevents neuronal atrophy [10].

Balancing the processes of fission and fusion controls the overall morphology and function of mitochondria, and numerous proteins that regulate fission and fusion have been reported [11]. Dynamin-related protein 1 (Drp1) is a central mitochondrial fission protein, recruited to fission sites on the outer mitochondrial membrane. Loss of Drp1 has been shown to deplete axonal mitochondria, which is followed by axonal energy failure and neuronal death [12]. When mitochondria are damaged, their membrane potential is reduced and Drp1-mediated fission and apoptotic signals are stimulated [13]. Mitochondrial division inhibitor-1 (Mdivi-1) was identified during chemical screening for mitochondrial division inhibitors; it selectively inhibits Drp1 function by inhibiting Drp1 self-assembly [14]. Parkin, a ubiquitin-protein ligase, has been shown to regulate mitochondrial morphology, and loss-of-function Parkin mutations produce enlarged or swollen mitochondria with suppressed Drp1-mediated fission [15].

In this study, we investigated Drp1 function during KA-induced excitotoxicity by using Mdivi-1 and suggest that inhibiting excessive mitochondrial fragmentation may have therapeutic benefit by protecting against neuronal death. We showed that Mdivi-1 significantly reduced seizure activity and increased survival of KA-treated mice. By reducing Parkin-mediated mitochondrial degradation, Mdivi-1 may protect against mitochondrial functional failure and neuronal death.

## Results

### Mdivi-1 reduces seizure activity and increases survival of mice with KA-induced seizures

**Fig. 1:**
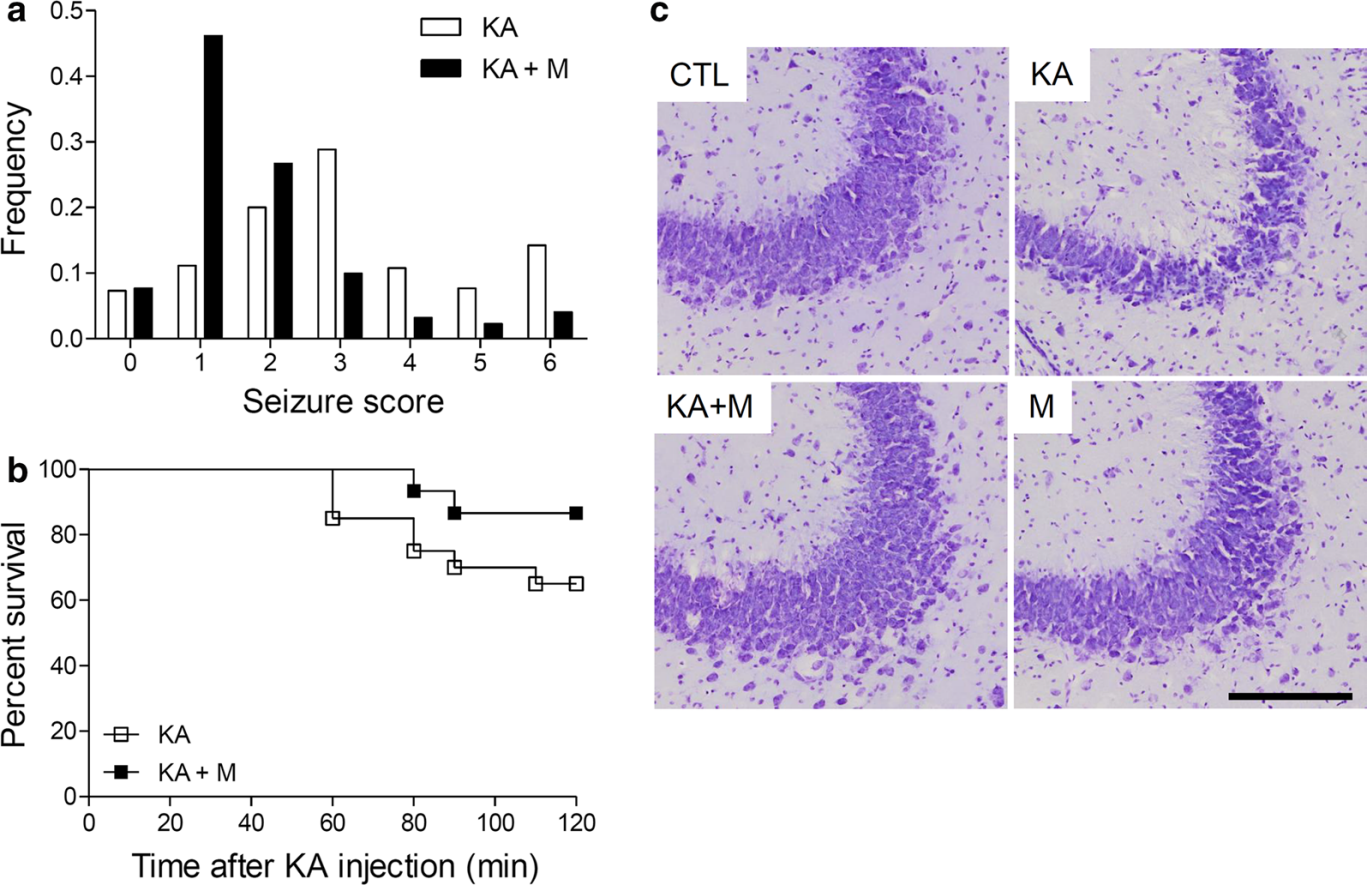
Mdivi-1 effects on KA-induced seizure activity, percent survival, and neuronal cell death. The frequency of behavioral seizure scores (**a**) and percent survival (**b**) were monitored during the first 2 h after KA treatment (*P* < 0.05 KA versus KA + M). **c** Cresyl violet-stained brain sections showing pyramidal neurons in the CA3 regions. The brain sections were prepared at 24 h after KA injection. *Scale bar* 200 µm

To determine whether Mdivi-1 has an effect on KA-induced seizures and survival, we monitored seizure activity and survival rates for 2 h after KA injection (Fig. 1a, b). KA-treated (KA) mice and Mdivi-1-treated (KA + M) mice showed different distributions in the frequency of behavioral seizure scores after KA injection (*P* < 0.05, Fig. 1a); the Chi square calculated value (χ^2^ = 106.41) was greater than the Chi square critical value (χ^2^* = 12.59) for *P* = 0.05. Mdivi-1 treatment reduced the frequency of seizure scores above 3 in KA-injected mice. The frequency of seizure scores 4–6 was 0.33 in the KA mice, whereas the frequency was 0.095 in the KA + M mice (*P* < 0.05). The mortality rate was 35 % (n = 7/20) in the KA mice and 12 % (n = 2/17) in the KA + M mice after KA injection (Fig. 1b). These results thus showed that Mdivi-1 reduces seizure activity and mortality in a mouse model of KA-induced seizures.

### Mdivi-1 attenuates neuronal cell death in the hippocampus after KA treatment

We subsequently used cresyl violet staining to examine whether Mdivi-1 protects KA mice from seizure-induced neuronal death (Fig. 1c). KA mice exhibited significant hippocampal cell death; the thickness of pyramidal cells in the CA3 region was reduced, typical pyknotic nuclei were found, and in some cases, there was a complete loss of hippocampal cells (Fig. 1c, Additional file 1: Figure S1A). The number of NeuN-positive cells was counted in the CA3 regions of CTL, KA, KA + M, and M mice and KA mice showed ~60 % cell loss compared to CTL mice (Additional file 1: Figure S1B). However, these changes were only partial or significantly reduced in the hippocampus of KA + M mice. These results thereby showed that Mdivi-1 attenuates KA-induced neuronal cell death.

### Mdivi-1 protects mitochondrial morphology in the hippocampus after KA treatment

**Fig. 2:**
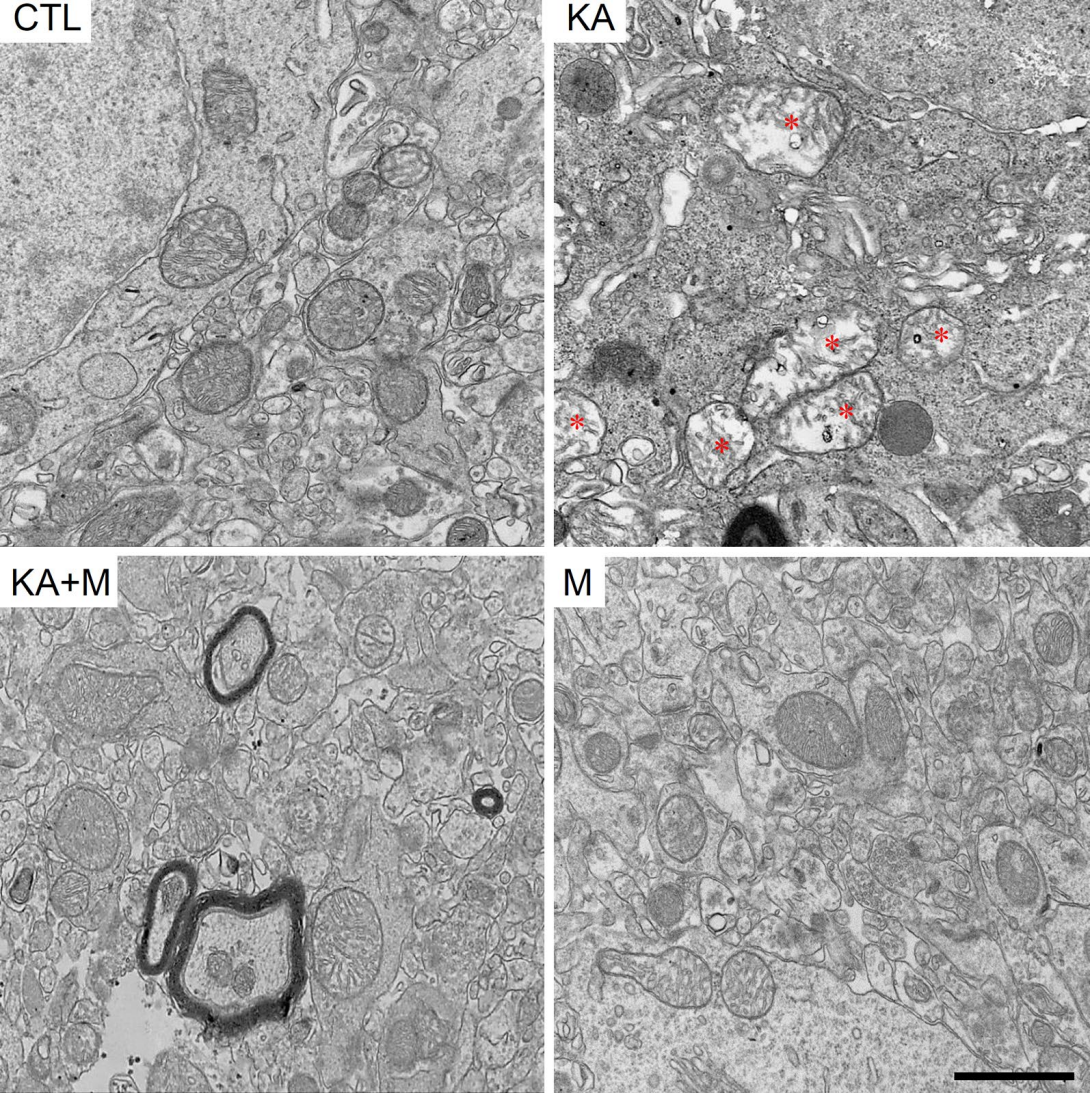
Mdivi-1 effects on mitochondrial morphology in the mouse hippocampus 24 h after KA injection. Representative electron microscopy images showing mitochondrial ultrastructure in the hippocampus of CTL, KA, KA + M, and M mice. *Asterisks* indicate the mitochondria with disrupted cristae structures. *Scale bar* 1 µm

Since Mdivi-1 is a mitochondrial fission inhibitor by regulating Drp1, we examined whether Mdivi-1 pretreatment protects neuronal death by affecting mitochondrial morphology in KA mice. Electron microscopy showed that KA induced significant changes in mitochondrial size and cristae structure (Fig. 2). In KA-injected mice, the number of mitochondria was significantly reduced and the area and perimeter were heterogeneous- some larger mitochondria had fragmented or completely absent cristae structure; these changes were attenuated by Mdivi-1 treatment (Additional file 1: Figure S2).

**Fig. 3:**
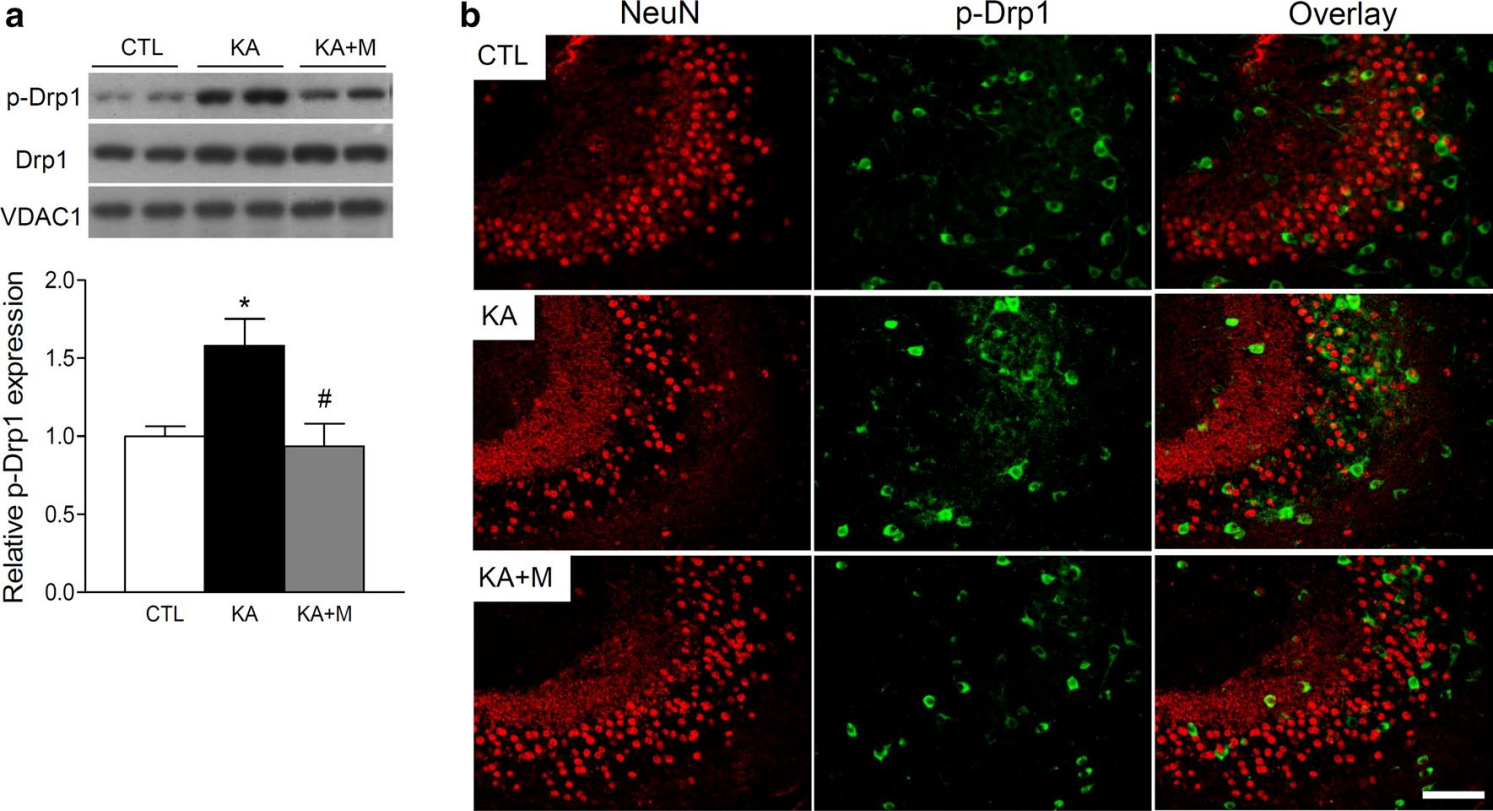
Mdivi-1 effects on mitochondrial p-Drp1 expression in the mouse hippocampus 24 h after KA injection. **a** Western blots and quantitative protein analysis of p-Drp1, Drp1, and VDAC1 in the hippocampal mitochondrial fractions from CTL, KA, and KA + M mice. Densitometry values were normalized to Drp1 and expressed as arbitrary units. Data are shown as mean ± SEM. *P < 0.05 versus CTL. ^#^ *P* < 0.05 versus KA. **b** Representative immunofluorescence images of NeuN, p-Drp1, and overlay in the hippocampus of CTL, KA, and KA + M mice. *Scale bar* 100 µm

To examine whether Mdivi-1 pretreatment changed Drp1 activity, the phosphorylation of Drp1 (p-Drp1) at Ser616, which is known to stimulate fission after phosphorylation [16], was assessed by Western blot and immunofluorescence staining analysis (Fig. 3). The expression of p-Drp1 was significantly increased in KA mice and reduced in KA + M mice (Fig. 3a). The intensity of p-Drp1 immunofluorescence staining in the hippocampal CA3 region was increased (~20 %) in KA mice compared to CTL mice and reversed after Mdivi-1 pretreatment (Fig. 3b). We also found that KA increased the number of p-Drp1 stained GABAergic interneurons in the hippocampal CA3 region (Additional file 1: Figure S3) that may indicate the impaired balance between excitation and inhibition in CA1 neuronal circuitry [17]. These results thus suggest that Mdivi-1 may block p-Drp1 mediated mitochondrial fragmentation and reduce significant principal neuronal loss after KA and further alter GABAergic inhibitory interneurons.

The balance between mitochondrial fusion and fission is also regulated by the activity of fusion proteins. Optic atrophy 1 (Opa1) and mitofusin (Mfn) are localized to the inner and outer membranes of the mitochondria, respectively, and both are required for fusion [11]. Thus, we examined whether the expression of Opa1 and Mfn2 was altered in KA mice. Western blot analysis showed that Opa1 and Mfn2 levels were similar among the groups of mice (Additional file 1: Figure S4A, B). In addition, there was no change in the expression of cyclophilin D (CypD), a matrix protein that modulates mitochondrial permeability transition pore (MPTP) opening (Additional file 1: Figure S4C). These results thereby suggest that Mdivi-1 regulates mitochondrial fission mainly by regulating Drp1 activity through reduced Ser616 phosphorylation to protect hippocampal neurons from KA-induced mitochondrial degradation and neuronal death.

### Effect of Mdivi-1 on Parkin and Hsp72 expression in the hippocampus after KA treatment

**Fig. 4:**
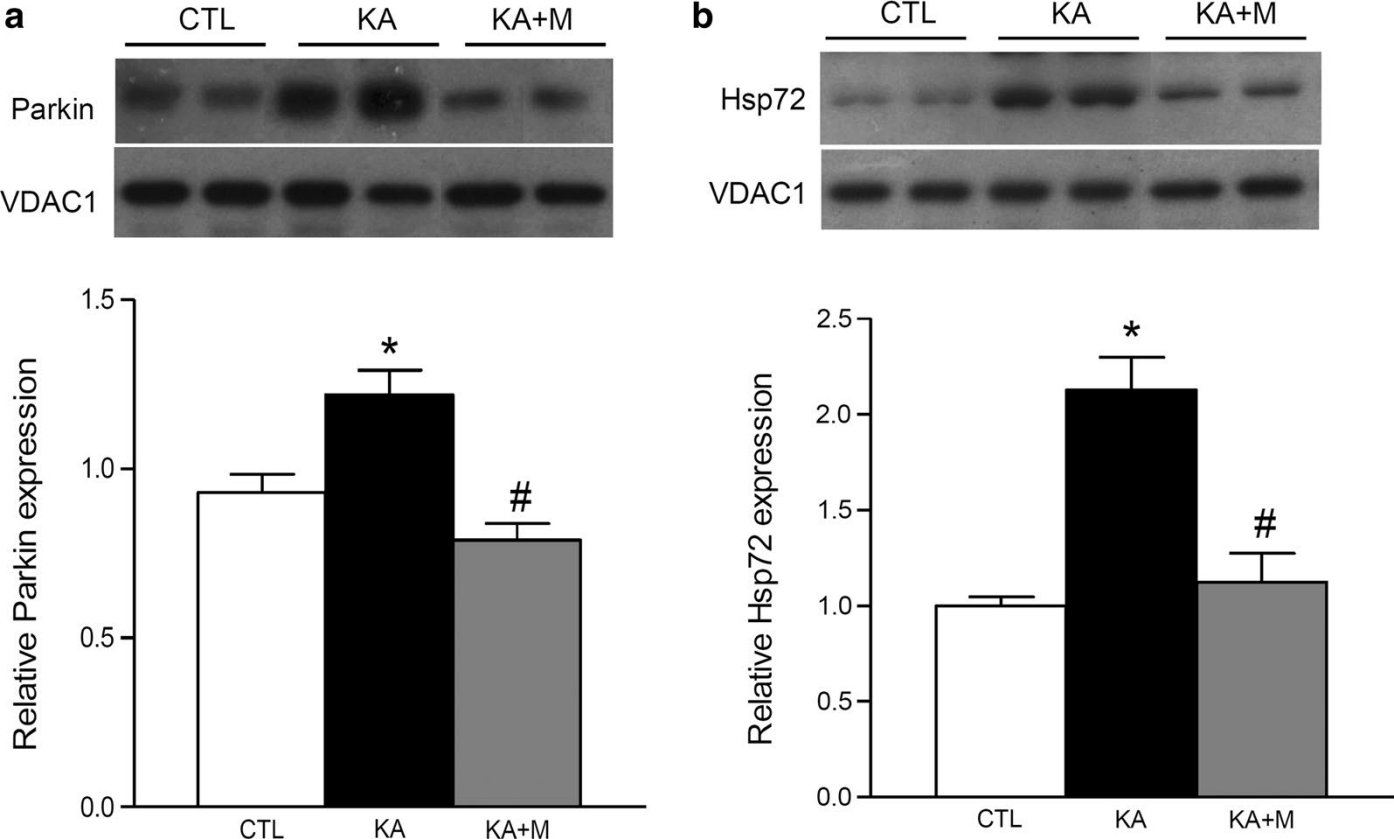
Mdivi-1 effects on mitochondrial Parkin and Hsp72 expression in the mouse hippocampus 24 h after KA injection. Western blots and quantitative protein analysis of Parkin (**a**) and Hsp72 (**b**) in the hippocampal mitochondrial fractions from CTL, KA, and KA + M mice. Densitometry values were normalized to VDAC1 and expressed as arbitrary units. Data are shown as mean ± SEM. *P < 0.05 versus CTL. ^#^ *P* < 0.05 versus KA

To examine the effect of Mdivi-1 on Parkin recruitment to the mitochondria of KA mice, we performed Western blot analysis. Parkin is normally localized in the cytoplasm and selectively recruited to impaired mitochondria where it promotes mitochondrial degradation [18]. To examine the subcellular localization of Parkin, cytoplasmic and mitochondrial fractions were isolated and Parkin levels were examined (Fig. 4a). Mitochondrial Parkin was induced by KA and significantly decreased by Mdivi-1 pretreatment. Next, we examined the mitochondrial expression of heat shock protein 72 (Hsp72), which is a stress sensor that translocates to damaged mitochondria and regulates Parkin-mediated mitophagy [19]. Mitochondrial Hsp72 expression was significantly induced in KA mice, and Mdivi-1 treatment reduced Hsp72 levels (Fig. 4b).

### Effects of Mdivi-1 treatment on hippocampal Cox-2 and Iba-1 expression

**Fig. 5:**
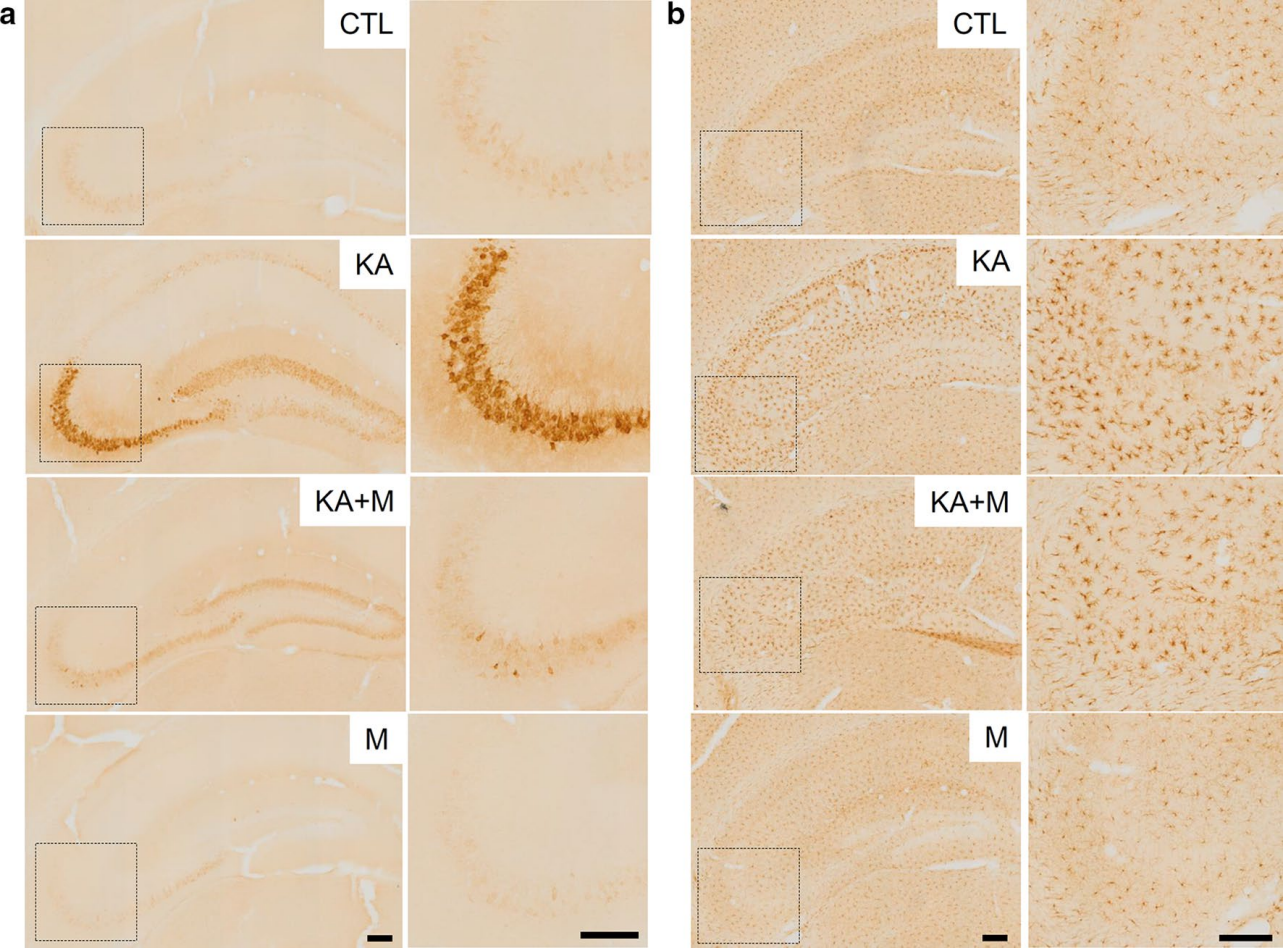
Mdivi-1 effects on Cox-2 and Iba-1 expression in the mouse hippocampus 24 h after KA injection. Immunohistochemistry of Cox-2 (**a**) and Iba-1 (**b**) in the hippocampus of CTL, KA, KA + M, and M mice. The CA3 regions are indicated as *dotted squares* and shown magnified at the *right*. *Scale bar* 200 µm

To evaluate whether Mdivi-1 treatment protects the hippocampus from KA-induced neuronal and glial inflammation, we examined expression of Cox-2 and Iba-1 in control (CTL), KA, and KA + M mice (Fig. 5). The expression of Cox-2 was dramatically increased in the hippocampal CA3 region of KA mice compared to the expression in CTL mice, and Mdivi-1 pretreatment reduced neuronal Cox-2 expression (Fig. 5a). Moreover, we examined microglial activation by Iba-1 expression in the hippocampus of CTL, KA, and KA + M mice. KA induced morphological changes in immune-responsive microglial cells and increased the number of activated microglial cells and the Iba-1 expression levels, which were significantly reduced by Mdivi-1 treatment (Fig. 5b, Additional file 1: Figure S5). These results suggest that Mdivi-1 protects the hippocampus from KA-induced inflammation by inhibiting mitochondrial fragmentation and subsequent dysfunction.

## Discussion

The present study showed that Mdivi-1 pretreatment attenuates seizure activity and prevents KA-induced hippocampal cell death by reducing mitochondrial morphological changes, mitochondrial fission, and Parkin-mediated degradation. We hypothesized that KA-induced excitotoxicity increases mitochondrial fission, leading to hippocampal neuronal death. We treated mice with a low dose of Mdivi-1 before KA injection to examine whether Mdivi-1 exhibited neuroprotective effects by modifying the mitochondrial stress response at an early stage of seizure pathology, and we demonstrated that Mdivi-1 ultimately reduced neuroinflammation and neuronal death in the hippocampus of KA-injected mice.

We first showed that Mdivi-1 pretreatment reduced seizure behaviors and increased mouse survival rates upon KA injection. Neuronal death in the hippocampal CA3 region was also significantly reduced in Mdivi-1 pretreated KA-injected mice. These findings are in agreement with previous reports demonstrating that Mdivi-1 protects against hippocampal neuronal death in pilocarpine-induced seizures in rats [20, 21]. Since Mdivi-1 is known to inhibit mitochondrial fission factor, Drp1, we further examined mitochondrial morphology via electron microscopy. The mitochondrial sizes were heterogeneous, and the mitochondrial inner and outer membrane structures were severely disrupted, suggesting that the mitochondrial membrane potential and other buffering functions of the mitochondria were impaired. Recently, Drp1 inhibition by Mdivi-1 in traumatic and ischemic brain injury was shown to reduce morphological and functional changes in mitochondria, cell apoptosis, neurological deficits, and brain edema [14, 22].

Here, we did not examine the chronic effect of Mdivi-1 at the time longer than 24 h after KA injection. The chronic changes include limbic sclerosis, a massive neurodegeneration and spontaneous recurrent seizure that are frequently shown in the developed epilepsy such as human temporal lobe epilepsy (TLE). These changes are often studied at 7–8 weeks after status epilepticus (SE) induction (KA injection in our model) [23], in that we may study the neuroprotective effect of Mdivi-1 from KA-induced chronic changes. In addition, we showed only mitochondrial morphological changes by EM analysis; however, we expect significant mitochondrial functional changes that can be accessed by a variety of experimental technologies which were limited in the current study. Future study to understand mitochondrial functional outcomes of the Mdivi-1 treatment would examine the changes in mitochondrial membrane potential, oxidative stress, calcium levels, ATP production, and oxidative phosphorylation.

Mitochondrial fission is controlled in part by the phosphorylation of Drp1; when phosphorylated at Ser616, Drp1 stimulates mitochondrial fission [16, 24]. We demonstrated that p-Drp1 levels were significantly increased in mitochondria isolated from the hippocampus of KA-injected mice and in co-localized CA3 neurons in KA-injected mice, whereas Mdivi-1 pretreatment inhibited the increase of p-Drp1 expression. Mitochondrial fission has been previously shown to promote apoptosis by increasing mitochondrial outer membrane permeability, and Mdivi-1 was shown to block Bax/Bak-dependent cytochrome C release from mitochondria [14, 22]. We also demonstrated that Parkin recruitment to mitochondria isolated from the hippocampus of KA-injected mice was increased, whereas Mdivi-1 inhibited this recruitment. Parkin has been previously shown to be selectively recruited to damaged mitochondria with low membrane potential, and it plays a role in mediating mitochondrial degradation [18]. Our results suggest that Mdivi-1 prevents mitochondrial fission and Parkin-mediated degradation in KA-injected mice.

Hsp72 is a critical regulator of stress-induced Parkin-mediated mitochondrial degradation, since Hsp72 rapidly translocates to depolarized mitochondria prior to Parkin recruitment. Furthermore, impaired Parkin action in Hsp72 knockout mice has been associated with altered mitochondrial morphology and reduced mitochondrial function, suggesting that Hsp72 may regulate mitochondrial quality control and have therapeutic benefit [19]. In our study, we showed that Mdivi-1 inhibited the increase of Hsp72 expression in mitochondria isolated from the hippocampus of KA-induced seizure mice, suggesting that Mdivi-1 prevents Parkin-mediated mitochondrial degradation.

Based on our findings, we suggest that inhibition of excessive mitochondrial fission induced by KA injection may reduce the initial mitochondrial excitotoxic damage response and subsequent hippocampal neuronal death. Our study was limited to examine the protein expression or mitochondrial changes at 24 h after KA injection and no data were provided on early time points when we may show the causal effect of initial mitochondrial changes on KA excitotoxcitiy following neuronal death in the hippocampus. We also examined changes in expression of Opa1 and Mfn2, two mitochondrial proteins involved in the regulation of mitochondrial fusion, and CypD, which modulates opening of MPTP [25, 26]. We observed no changes in expression levels for any of these proteins upon KA injection or Mdivi-1 pretreatment. Thus, we assume that the dose of Mdivi-1 administered to KA mice in the present study affected mitochondrial fission through Drp1 and Parkin-mediated mitophagy, but not through other mitochondrial regulatory factors in at least the time points we examined.

In a recent study, Mdivi-1 showed a neuroprotective effect in a mouse model of traumatic brain injury (TBI), where Mdivi-1 alleviated TBI-induced behavioral deficits and brain edema, and neuronal death. The underlying mechanism may be associated with reducing mitochondrial morphological changes, inhibiting mitochondrial fission, cytochrome c release and caspase-3 activation [27]. In addition, stimulating mitochondrial biogenesis by peroxisome proliferator-activated receptor gamma coactivator-1α (PGC-1α) was shown to be neuroprotective in KA-induced neuronal death by increasing anti-apoptotic protein and mitochondrial number and function of oxidative phosphorylation [28]. These studies support that normalizing mitochondrial morphology and the number, and enhancing mitochondrial function by Mdivi-1 are beneficial against neurodegenerative diseases.

During the study, we found that KA induced p-Drp1 expression in GABAergic inhibitory neurons and Mdivi-1 treatment reduced the level. We suggest that the p-Drp1-mediated mitochondrial dysfunction may alter inhibitory GABAergic neural transmission in KA mice and lead to chronic seizures by disrupting the balance between excitation and inhibition in CA1 neuronal circuitry [17], which may be attenuated by Mdivi-1 treatment.

## Conclusions

In the current study, we demonstrate the neuroprotective effect of Mdivi-1, which supports the suggestion that correcting mitochondrial morphology may reduce the extent of neuroinflammation and neuronal death induced by seizures. The molecular mechanism of Mdivi-1 involves attenuating mitochondrial fission through Drp1 and Hsp72/Parkin-mediated mitochondrial degradation. Mdivi-1 is a promising target for the treatment of seizures, strokes, other neurotoxic and/or ischemic damage, as well as neurodegenerative diseases.

## Methods

### Animals

Male ICR mice (4-weeks old) were purchased from KOATECH Co. (Pyeongtaek, South Korea) and maintained in the animal facility at Gyeongsang National University (GNU). All animal experiments were approved by the Institutional Board of Animal Research at GNU and performed in accordance with the National Institutes of Health guidelines for laboratory animal care (GNU-140922-M0046). Mice were individually housed with exposure to an alternating 12-h light/dark cycle and were provided with water and standard chow ad libitum.

### Drug treatment and seizure induction

The mice were divided randomly into four groups: saline-injected mice (CTL, n = 14), KA (Abcam, Cambridge, MA, USA)-injected mice (KA, n = 20), mice injected with Mdivi-1 (Sigma-Aldrich, St. Louis, MO, USA) and KA (KA + M, n = 17), and mice injected with Mdivi-1 and saline (M, n = 8). The KA + M mice received an intraperitoneal (i.p.) injection of 20 mg/kg Mdivi-1 emulsified in dimethyl sulfoxide (DMSO) twice, at 24 h and 30 min before the injection of KA [22, 29]. Mice were then given an i.p. injection of 30 mg/kg KA emulsified in 0.9 % normal saline. Control mice received i.p. injections of DMSO or saline at the same time. After KA injection, the mice were monitored for 2 h for the onset and extent of seizure activity. Seizure activity was quantitated using a six-point seizure scale, as previously described [30]. Only those mice that developed seizures after KA were used for further examination. Once a seizure was confirmed, the mice in each group were sacrificed at 24 h after KA treatment.

### Tissue preparation and cresyl violet staining

Mice (CTL, n = 5; M, n = 3; KA, n = 5; KA + M, n = 5) were anesthetized with zoletil (5 mg/kg; Virbac Laboratories, Carros, France), perfused transcardially with heparinized saline, and their brains were fixed using 4 % paraformaldehyde (PFA) in 0.1 M phosphate-buffered saline (PBS). For regular immunostaining, the brains were post-fixed for 6 h in 4 % PFA fixative and then sequentially immersed in 0.1 M PBS containing 15 % sucrose and PBS containing 30 % sucrose at 4 °C until the tissues sank to the bottom of the container. After being frozen in OCT compound (Sakura Finetek USA Inc., Torrance, CA, USA), the brains were cut into 40-µm thick coronal sections. The sections were stained with cresyl violet, and visualized with a BX51 microscope (Olympus, Tokyo, Japan). Digital images were captured.

## Electron microscopy

The mice (n = 2 each group) were anesthetized, perfused with saline, and fixed using 4 % PFA in 0.1 M phosphate buffer (PB). The brains were sectioned into 1-mm thick slices, then stored in a mixture of 2.5 % glutaraldehyde in 0.1 M PB at 4 °C before further processing, as previously described [31]. Images were obtained by a transmission electron microscope (Carl Zeiss, LIBRA 120, Oberkochen, Germany) at 120 kV. The morphological properties of mitochondria (area and perimeter) were quantified by using Image J (National Institutes of Health, Bethesda, MD, USA, http://imageJ.nih.gov/ij).

### Immunohistochemistry

Frozen free-floating brain sections were incubated with primary antibodies (Cox-2 from Cayman 160126 and Iba-1 from Wako 019-19741) overnight at 4 °C. After washing three times with 0.1 M PBS, the sections were incubated for 1 h at room temperature with biotinylated secondary antibodies. After washing three times with 0.1 M PBS, the sections were incubated in avidin–biotin-peroxidase complex solution (ABC solution; Vector Laboratories, Burlingame, CA, USA). The sections were then developed using a DAB (3,3′-diaminobenzidine) Peroxidase Substrate Kit (Vector Laboratories, Inc., Burlingame, CA, USA), mounted on gelatin-coated slides, warm-dried, dehydrated using a gradient of alcohol solutions, cleared in xylene, and mounted on coverslips with Permount (Sigma-Aldrich). The sections were visualized with a BX51 microscope (Olympus) and digital images were captured. The intensity of ba-1 expression was quantified by using Image J (NIH) and the number of activated microglial cells that showed morphological changes (long branched processes and increased cell body) in the CA3 regions was counted in 4–5 fields per single slice (at the same level) from each group.

### Immunofluorescence

For double immunostaining, free-floating sections were incubated with primary antibodies (NeuN from Millipore MAB377, p-Drp1(Ser616) from Cell Signaling #3455, and GABA Rα1 from Santa Cruz: sc-31405) overnight at 4 °C. After washing three times with 0.1 M PBS, the sections were incubated with Alexa Fluor488- and/or 594-conjugated secondary antibodies (Invitrogen Life Technologies, Carlsbad, CA, USA). Fluorescence was visualized with a BX51 microscope (Olympus) and Metamorph image analysis (Molecular Devices, Sunnyvale, CA, USA) was used. The number of NeuN-positive cells was counted from 3 fields per single slice (at the same level) in the CA3 regions of both hippocampi from each group.

### Mitochondrial isolation and Western blot analyses

The mice were anesthetized and their brains (CTL, n = 7; M, n = 3; KA, n = 6, KA + M; n = 8) were quickly removed from the skull and both hippocampi were dissected and frozen. For mitochondrial isolation, the frozen hippocampi were transferred to a microcentrifuge tube containing 200 µL of mitochondria buffer (MB: sucrose 250 mM, KCl 10 mM, MgCl_2_ 5 mM, ethylenediaminetetraacetic acid (EDTA) 1 mM, ethylene glycol tetraacetic acid (EGTA) 1 mM, HEPES 20 mM) with protease inhibitors (Sigma-Aldrich). Tissues were homogenized and centrifuged at 500×*g* at 4 °C for 5 min, and the supernatant was transferred to a clean tube. To separate cytosolic and mitochondrial fractions, the supernatant was centrifuged at 10,000×*g* at 4 °C for 5 min, and the mitochondrial pellet was washed and resuspended in MB. The protein concentration was quantified with the Bio-Rad protein assay kit (Bio-Rad, Hercules, CA, USA), and samples were stored at −80 °C. Proteins were separated via sodium dodecyl sulphate polyacrylamide gel electrophoresis (SDS-PAGE), followed by electrophoretic transfer onto a polyvinylidene difluoride membrane (PVDF) (Millipore, Billerica, MA, USA). Proteins were subjected to immunoblotting with the primary antibodies (p-Drp1(Ser616) from Cell Signaling #3455, Parkin from Abcam 15954, Hsp72 from Enzo ADI-SPA-812, Opa1 from BD 612606, Mfn2 from Abcam 56889, CypD from Thermo Scientific PA3-022, voltage-dependent anion channel 1 (VDAC1) from Abcam 15895, and β-actin from Sigma-Aldrich A5441) and visualized with ECL substrates (Pierce, Rockford, IL, USA). The Multi Gauge V 3.0 image analysis program (Fujifilm, Tokyo, Japan) was used to measure the densitometry of protein bands.

### Statistical analyses

Statistical differences among groups of mice treated with different types of substances (vehicle, KA, and Mdivi-1) were determined using one-way analysis of variance (ANOVA), followed by Bonferroni post hoc analysis. Student’s *t* test was used when only two groups were compared. For comparison of seizure score data between KA and KA + M mice, the Chi square test was used. Statistical analysis was performed using Graphpad prism 5.04 (La Jolla, CA, USA) and dBSTAT 5.0 (Seoul, Korea). The experiments were repeated three times and the values are expressed as mean ± standard error of the mean (SEM). A *P* value <0.05 was considered statistically significant.

## Additional file

10.1186/s12868-016-0270-y A crystal violet-stained brain section (**A**) and the number of NeuN-positive cells in the CA3 region of CTL, KA, KA+M, and M mice (**B**). **Figure S2**. Morphological properties of mitochondria in the CA3 regions of CTL, KA, KA+M, and M mice; mitochondrial area and perimeter (**A**) and mitochondrial number/field and percentage of deformed mitochondria (**B**). **Figure S3**. Immunoflourescence images showing the GABA receptor α-1 and p-Drp1 in the CA3 regions (**A**) and the percentage of p-Drp1 and GABA-positive cells (**B**). **Figure S4**. Mdivi-1 effects on mitochondrial OPA1, Mfn2, and CypD expression in the hippocampus 24h after KA injection.  **Figure S5**. Iba-1 expression levels (**A**) and the number of activated microglial cells (**B**) in the CA3 regions of CTL, KA, KA+M, and M mice.

## Abbreviations

Cox-2: cyclooxygenase-2
CTL: control
CypD: cyclophilin D
EM: electron microscopy
GABA: γ-aminobutyric acid
Hsp72: heat shock protein 72
KA: kainic acid
Mdivi-1: mitochondrial division inhibitor-1
Mfn: mitofusin
MPTP: mitochondrial permeability transition pore
SEM: standard error of mean
VDAC1: voltage-dependent anion channel 1

## References

[1] Korhonen L, Belluardo N, Lindholm D (2001). Regulation of X-chromosome-linked inhibitor of apoptosis protein in kainic acid-induced neuronal death in the rat hippocampus. Mol Cell Neurosci.

[2] Olney JW, Rhee V, Ho OL (1974). Kainic acid: a powerful neurotoxic analogue of glutamate. Brain Res.

[3] Dong XX, Wang Y, Qin ZH (2009). Molecular mechanisms of excitotoxicity and their relevance to pathogenesis of neurodegenerative diseases. Acta Pharmacol Sin.

[4] Filosto M, Scarpelli M, Cotelli MS, Vielmi V, Todeschini A, Gregorelli V, Tonin P, Tomelleri G, Padovani A (2011). The role of mitochondria in neurodegenerative diseases. J Neurol.

[5] Johri A, Beal MF (2012). Mitochondrial dysfunction in neurodegenerative diseases. J Pharmacol Exp Ther.

[6] Chuang YC, Chang AY, Lin JW, Hsu SP, Chan SH (2004). Mitochondrial dysfunction and ultrastructural damage in the hippocampus during kainic acid-induced status epilepticus in the rat. Epilepsia.

[7] Kudin AP, Kudina TA, Seyfried J, Vielhaber S, Beck H, Elger CE, Kunz WS (2002). Seizure-dependent modulation of mitochondrial oxidative phosphorylation in rat hippocampus. Eur J Neurosci.

[8] Kunz WS, Kudin AP, Vielhaber S, Blumcke I, Zuschratter W, Schramm J, Beck H, Elger CE (2000). Mitochondrial complex I deficiency in the epileptic focus of patients with temporal lobe epilepsy. Ann Neurol.

[9] Youle RJ, van der Bliek AM (2012). Mitochondrial fission, fusion, and stress. Science.

[10] Chen H, Chan DC (2009). Mitochondrial dynamics–fusion, fission, movement, and mitophagy–in neurodegenerative diseases. Hum Mol Genet.

[11] Scott I, Youle RJ (2010). Mitochondrial fission and fusion. Essays Biochem.

[12] Verstreken P, Ly CV, Venken KJ, Koh TW, Zhou Y, Bellen HJ (2005). Synaptic mitochondria are critical for mobilization of reserve pool vesicles at Drosophila neuromuscular junctions. Neuron.

[13] Grohm J, Kim SW, Mamrak U, Tobaben S, Cassidy-Stone A, Nunnari J, Plesnila N, Culmsee C (2012). Inhibition of Drp1 provides neuroprotection in vitro and in vivo. Cell Death Differ.

[14] Cassidy-Stone A, Chipuk JE, Ingerman E, Song C, Yoo C, Kuwana T, Kurth MJ, Shaw JT, Hinshaw JE, Green DR (2008). Chemical inhibition of the mitochondrial division dynamin reveals its role in Bax/Bak-dependent mitochondrial outer membrane permeabilization. Dev Cell.

[15] Poole AC, Thomas RE, Andrews LA, McBride HM, Whitworth AJ, Pallanck LJ (2008). The PINK1/Parkin pathway regulates mitochondrial morphology. Proc Natl Acad Sci USA.

[16] Qi X, Disatnik MH, Shen N, Sobel RA, Mochly-Rosen D (2011). Aberrant mitochondrial fission in neurons induced by protein kinase Cδ under oxidative stress conditions in vivo. Mol Biol Cell.

[17] Liu YQ, Yu F, Liu WH, He XH, Peng BW (2014). Dysfunction of hippocampal interneurons in epilepsy. Neurosci Bull.

[18] Narendra D, Tanaka A, Suen DF, Youle RJ (2008). Parkin is recruited selectively to impaired mitochondria and promotes their autophagy. J Cell Biol.

[19] Drew BG, Ribas V, Le JA, Henstridge DC, Phun J, Zhou Z, Soleymani T, Daraei P, Sitz D, Vergnes L (2014). HSP72 is a mitochondrial stress sensor critical for Parkin action, oxidative metabolism, and insulin sensitivity in skeletal muscle. Diabetes.

[20] Qiu X, Cao L, Yang X, Zhao X, Liu X, Han Y, Xue Y, Jiang H, Chi Z (2013). Role of mitochondrial fission in neuronal injury in pilocarpine-induced epileptic rats. Neuroscience.

[21] Xie N, Wang C, Lian Y, Zhang H, Wu C, Zhang Q (2013). A selective inhibitor of Drp1, mdivi-1, protects against cell death of hippocampal neurons in pilocarpine-induced seizures in rats. Neurosci Lett.

[22] Zhao YX, Cui M, Chen SF, Dong Q, Liu XY (2014). Amelioration of ischemic mitochondrial injury and Bax-dependent outer membrane permeabilization by Mdivi-1. CNS Neurosci Ther.

[23] Brandt C, Potschka H, Loscher W, Ebert U (2003). *N*-methyl-d-aspartate receptor blockade after status epilepticus protects against limbic brain damage but not against epilepsy in the kainate model of temporal lobe epilepsy. Neuroscience.

[24] Knott AB, Perkins G, Schwarzenbacher R, Bossy-Wetzel E (2008). Mitochondrial fragmentation in neurodegeneration. Nat Rev Neurosci.

[25] Li V, Brustovetsky T, Brustovetsky N (2009). Role of cyclophilin D-dependent mitochondrial permeability transition in glutamate-induced calcium deregulation and excitotoxic neuronal death. Exp Neurol.

[26] Schinzel AC, Takeuchi O, Huang Z, Fisher JK, Zhou Z, Rubens J, Hetz C, Danial NN, Moskowitz MA, Korsmeyer SJ (2005). Cyclophilin D is a component of mitochondrial permeability transition and mediates neuronal cell death after focal cerebral ischemia. Proc Natl Acad Sci USA.

[27] Wu Q, Xia SX, Li QQ, Gao Y, Shen X, Ma L, Zhang MY, Wang T, Li YS, Wang ZF (2016). Mitochondrial division inhibitor 1 (Mdivi-1) offers neuroprotection through diminishing cell death and improving functional outcome in a mouse model of traumatic brain injury. Brain Res.

[28] Makela J, Mudo G, Pham DD, Di Liberto V, Eriksson O, Louhivuori L, Bruelle C, Soliymani R, Baumann M, Korhonen L (2016). Peroxisome proliferator-activated receptor-gamma coactivator-1alpha (PGC-1alpha) mediates neuroprotection against excitotoxic brain injury in transgenic mice—role of mitochondria and X-linked inhibitor of apoptosis protein. Eur J Neurosci.

[29] Rappold PM, Cui M, Grima JC, Fan RZ, de Mesy-Bentley KL, Chen L, Zhuang X, Bowers WJ, Tieu K (2014). Drp1 inhibition attenuates neurotoxicity and dopamine release deficits in vivo. Nat Commun.

[30] Jeong EA, Jeon BT, Shin HJ, Kim N, Lee DH, Kim HJ, Kang SS, Cho GJ, Choi WS, Roh GS (2011). Ketogenic diet-induced peroxisome proliferator-activated receptor-gamma activation decreases neuroinflammation in the mouse hippocampus after kainic acid-induced seizures. Exp Neurol.

[31] Cho SJ, Yun SM, Jo C, Lee DH, Choi KJ, Song JC, Park SI, Kim YJ, Koh YH (2015). SUMO1 promotes Aβ production via the modulation of autophagy. Autophagy..

