# Erratum to: A mitochondrial division inhibitor, Mdivi-1, inhibits mitochondrial fragmentation and attenuates kainic acid-induced hippocampal cell death

**DOI:** 10.1186/s12868-017-0339-2

**Published:** 2017-01-23

**Authors:** Hwajin Kim, Jong Youl Lee, Keon Jae Park, Won-Ho Kim, Gu Seob Roh

**Affiliations:** 1 Department of Anatomy and Convergence Medical Science, Institute of Health Sciences, Gyeongsang National University School of Medicine, 15, 816 Beon-gil, Jinju-daero, Jinju, Gyeongnam 660-751 Republic of Korea; 2 Division of Metabolic Diseases, Center for Biomedical Sciences, Korea National Institute of Health, Osong, Republic of Korea

## Erratum to: BMC Neurosci (2016) 17:33 DOI 10.1186/s12868-016-0270-y

In this version of this article that was originally published [1], there is incorrect funding information.

The funding information is currently:

This study was supported by a Grant of the Basic Science Research Program through the National Research Foundation (NRF) of Korea (Nos. 2014-0420 and 2015R1A5A2008833).

However, the number 2014-0420 is incorrect and should be 2014R1A1A1003211.
