# Supplementary material for: A mitochondrial division inhibitor, Mdivi-1, inhibits mitochondrial fragmentation and attenuates kainic acid-induced hippocampal cell death
Source: BMC Neurosci. 2016 Jun 10;17:33. doi: 10.1186/s12868-016-0270-y (PMC4902937; doi:10.1186/s12868-016-0270-y)

**Figure S1.** (A) A cresyl violet-stained brain section showing pyramidal neurons in the CA3 region of KA mice. Scale bar = 200 µm. (B) The number of NeuN-positive cells in the CA3 regions of CTL, KA, KA+M, and M mice (shown as percentage, CTL as 100%). Data are shown as mean ± SEM. **P* < 0.05 versus CTL. #*P* < 0.05 versus KA.

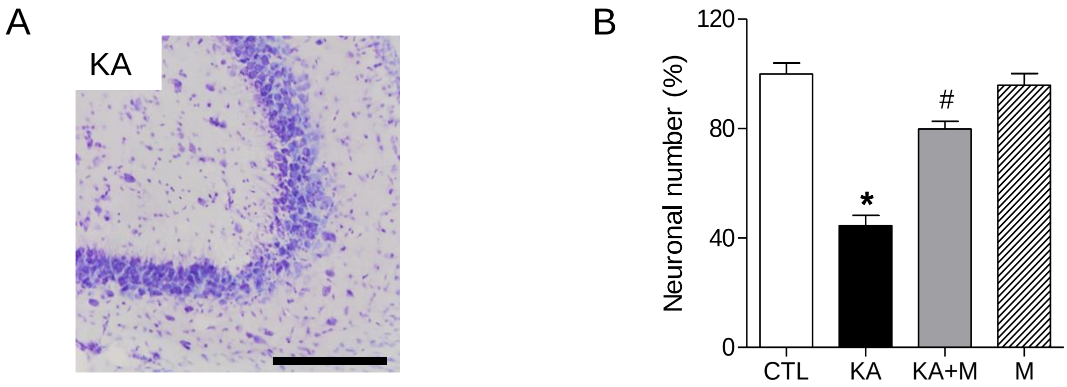

**Figure S2.** Morphological properties of mitochondria in the CA3 regions of CTL, KA, KA+M, and M mice. (A) Mitochondrial area and perimeter. (B) Mitochondrial number/field and percentage of deformed mitochondria out of total mitochondria analyzed. Data are shown as mean ± SEM. *$P < 0.05$ versus CTL. #$P < 0.05$ versus KA.

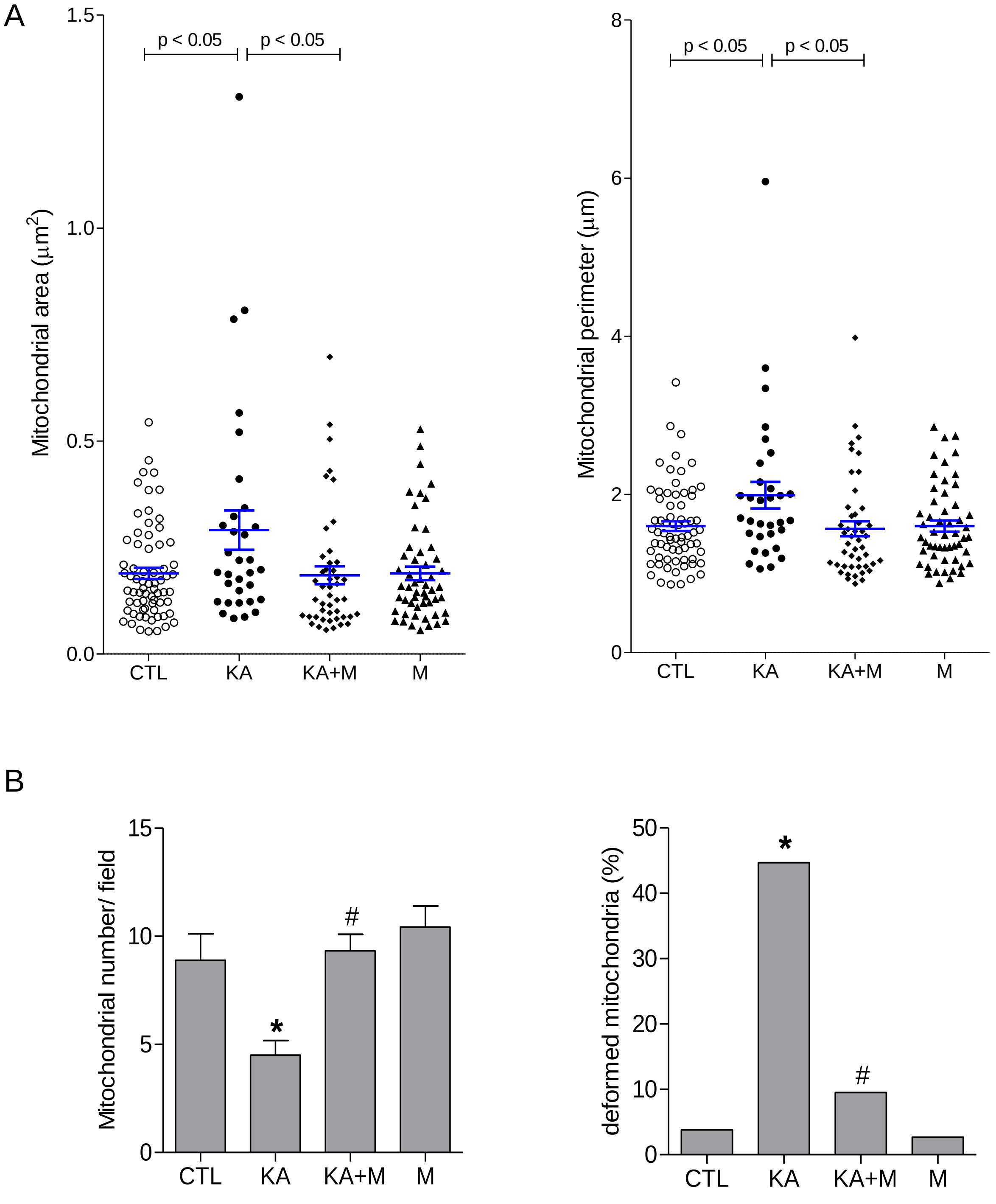

**Figure S3.** (A) Immunofluorescence images showing the stained cells by antibodies of GABA receptor α1 (red) and p-Drp1 (green) in the CA3 regions of CTL, KA, KA+M, and M mice. Scale bar = 100 μm. (B) The percentage of p-Drp1 and GABA-positive cells. Data are shown as mean ± SEM. *$P < 0.05$ versus CTL. $^{\#}P < 0.05$ versus KA.

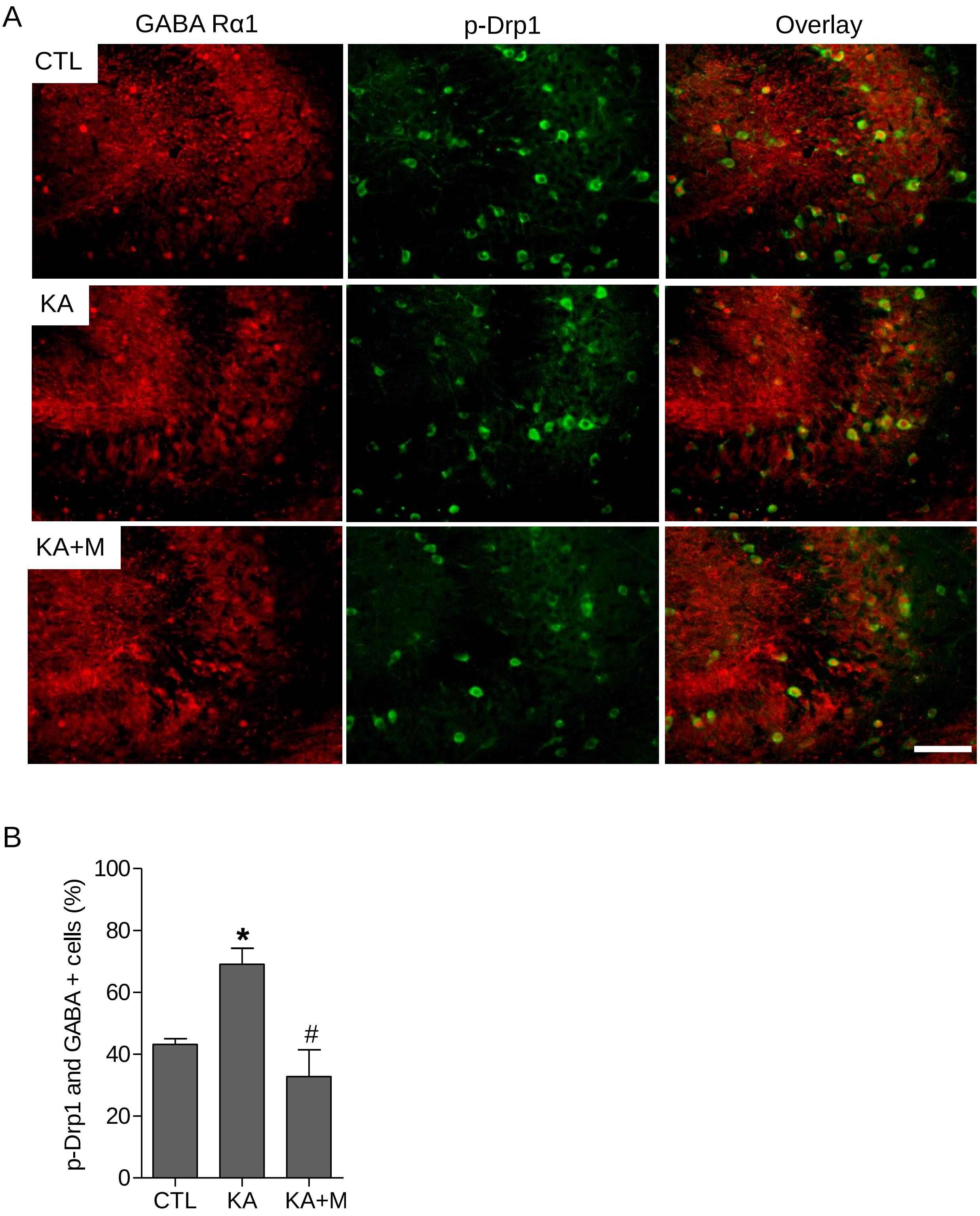

**Figure S4.** Mdivi-1 effects on mitochondrial OPA1, Mfn2, and CypD expression in the mouse hippocampus 24 h after KA injection. Western blots and quantification of OPA1 (A), Mfn2 (B), and CypD (C) in the hippocampal mitochondrial fractions from CTL, KA, and KA+M mice. Densitometry values were normalized to VDAC1 and expressed as arbitrary units. Data are shown as mean ± SEM. *$P$ < 0.05 versus CTL. $^{\#}P$ < 0.05 versus KA.

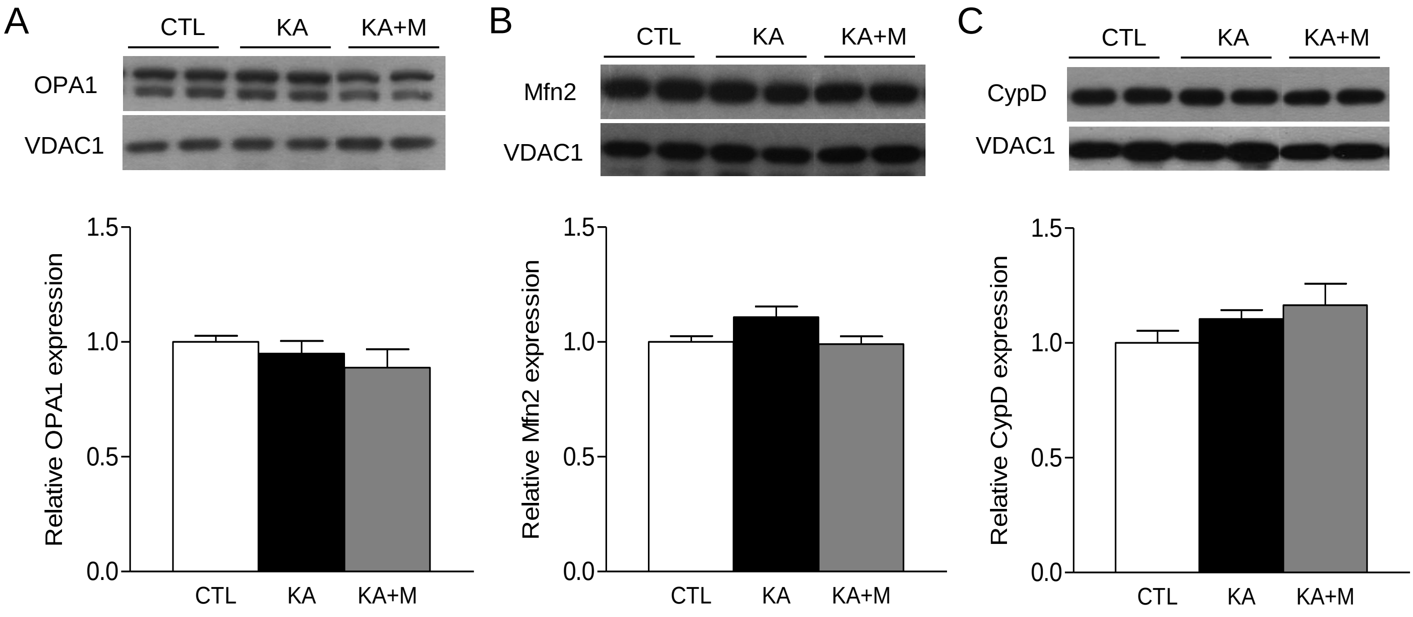

**Figure S5.** (A) Iba-1 expression levels in the CA3 regions of CTL, KA, KA+M, and M mice. (B) The number of activated microglial cells in the CA3 regions of CTL, KA, KA+M, and M mice (4~5 fields per each brain section). Data are shown as mean ± SEM. **P* < 0.05 versus CTL. #*P* < 0.05 versus KA.

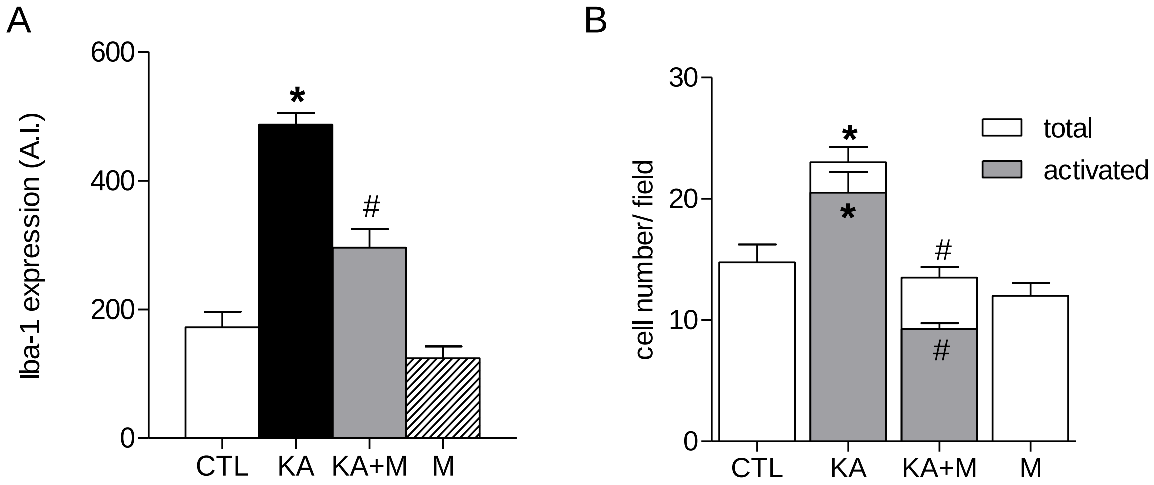

Supplement: Supplementary file 1 — 10.1186/s12868-016-0270-y A crystal violet-stained brain section (A) and the number of NeuN-positive cells in the CA3 region of CTL, KA, KA+M, and M mice (B). Figure S2. Morphological properties of mitochondria in the CA3 regions of CTL, KA, KA+M, and M mice; mitochondrial area and perimeter (A) and mitochondrial number/field and percentage of deformed mitochondria (B). Figure S3. Immunoflourescence images showing the GABA receptor α-1 and p-Drp1 in the CA3 regions (A) and the percentage of p-Drp1 and GABA-positive cells (B). Figure S4. Mdivi-1 effects on mitochondrial OPA1, Mfn2, and CypD expression in the hippocampus 24h after KA injection. Figure S5. Iba-1 expression levels (A) and the number of activated microglial cells (B) in the CA3 regions of CTL, KA, KA+M, and M mice. [file 12868_2016_270_MOESM1_ESM.pdf]
